# Mortality trends for ischemic heart disease in China: an analysis of 102 continuous disease surveillance points from 1991 to 2009

**DOI:** 10.1186/s12889-017-4558-3

**Published:** 2017-07-25

**Authors:** Xia Wan, Hongyan Ren, Enbo Ma, Gonghuan Yang

**Affiliations:** 1 Institute of Basic Medical Sciences, Chinese Academy of Medical Sciences and School of Basic Medicine, Peking Union Medical College, No. 5 Dong Dan San Tiao, Dongcheng District, Beijing, 100005 China; 2 State Key Laboratory of Resources and Environmental Information System, Institute of Geographic Sciences and Natural Resources Research, Chinese Academy of Sciences, No.11 Datuan Load A, Chaoyang District, Beijing, 100101 China; 3 Faculty of Medicine, University of Tsukuba, 1-1-1 Tennodai, Tsukuba, 305-8575 Japan

**Keywords:** Ischemic heart disease, Mortality rate, Disease surveillance points

## Abstract

**Background:**

In the past 20 years, the trends of ischemic heart disease (IHD) mortality in China have been described in divergent claims. This research analyzes mortality trends for IHD by using the data from 102 continuous Disease Surveillance Points (DSP) from 1991 to 2009.

**Method:**

The 102 continuous DSP covered 7.3 million people during the period 1991–2000, and then were expanded to a population of 52 million in the same areas for 2004–2009. The data were adjusted by using garbage code redistribution and underreporting rate, mapped from international classification of diseases ICD-9 to ICD-10. The mortality rates for IHD were further adjusted by the crude death proportion multiplied by the total number of deaths in the mortality envelope, which was calculated by using logr_t_ = a + bt. Age-standard death rates (ASDRs) were computed using China’s 2010 census population structure. Trend in IHD was calculated from ASDRs by using a joinpoint regression model.

**Results:**

The IHD ASDRs increased in total in regions with an average annual percentage change (AAPC) 4.96%, especially for the Southwest (AAPC = 7.97%) and Northeast areas (AAPC = 7.10%), and for male and female subjects (with 5% AAPC) as well. In rural areas, the year 2000 was a cut-off point for mortality rate with annual percentage change increasing from 3.52% in 1991–2000 to 9.02% in 2000–2009, which was much higher than in urban areas (AAPC = 1.05%). And the proportion of deaths increased in older adults, and more male deaths occurred before age 60 compared to female deaths.

**Conclusion:**

By observing a wide range of areas across China from 1991 to 2009, this paper concludes that the ASDR trend for IHD increased. These trends reflect changes in the Chinese standard of living and lifestyle with diets higher in fat, higher blood lipids and increased body weight.

**Electronic supplementary material:**

The online version of this article (doi:10.1186/s12889-017-4558-3) contains supplementary material, which is available to authorized users.

## Background

Ischemic heart disease (IHD) was the leading cause of death globally in the past two decades. In 2013, it caused as many deaths as chronic obstructive pulmonary disease (COPD), diabetes, cirrhosis, lung cancer, and liver cancer combined [1]. The mortality trend of IHD is one of the key indicators closely related to the global target of prevention and control of non-communicable diseases [2].

China, with its rapid economic development over the past 20 years, is experiencing a huge disease burden caused by cardiovascular diseases [3]. In 2012, cardiovascular disease (CVD) claimed 3.5 million lives in China, 40% of total deaths [4]. In China, there are two national surveillance systems, one is Disease Surveillance Points (DSP) system administered by Chinese Center for Disease Control & Prevention (CCDC) and the other is the sample vital registration (VR) system administered by National Health and Family Planning Commission (NHFPC) of the People’s Republic of China. *The Report on Cardiovascular Diseases in China (2016,* [5] showed that the IHD mortality rate increased from 2002 to 2015 by using data from NHFPC. Most studies from DSP also showed that the trend of age-standardized death rate (ASDR) for IHD in China has been increasing [6, 7]. Even in some cities of China, such as Beijing [8] and Tianjin [9], where the prevention strategies for CVD were implemented relatively well, the mortality rates of IHD still increased. But two newly published Global Burden of Disease (GBD) studies showed diverse results. GBD 2010 results showed that in China the ASDR for IHD increased from 55.7 per 100,000 in 1990 to 70.1 per 100,000 in 2010 [3]. While GBD 2013, using the same data sources, showed a different mortality trend for IHD in China, which remained stable over the past two decades, with 115.40 per 100,000 in 1990 and 115.89 per 100,000 in 2013 [10]. So, over the past 20 years in China, has the mortality rate for IHD increased or has it leveled off? Someone argued that the different mortality trends in IHD in the two studies is owing to covering different populations with adjustment to the disease surveillance system since 2000 [11].

In order to provide further evidence and determine the IHD mortality trends for policy-makers to create timely strategies for CVD prevention and control, this study examined the distribution and trends of IHD mortality in modern China by sex, age, area of residence (urban versus rural), and seven regions (Northeast, North, East, South, Central, Northwest and Southwest) of China, based on a relatively fixed population group for 102 continuous DSP during the past 20 years.

## Methods

### Data sources

The DSP system for recording causes of death was established in China in 1990, covering a population of 10 million people in 145 locations in all provinces by utilizing multiple-stratified random sampling [12]. The system was expanded in 2001 to cover 71.4 million people. The expanded DSP system was adjusted to cover 161 locations which included 103 of the original locations and 58 new locations [11]. The adjusted DSP system expanded to cover the whole district of a city or county, instead of one or two residential district(s) or town(s) at each location.

**Fig. 1:**
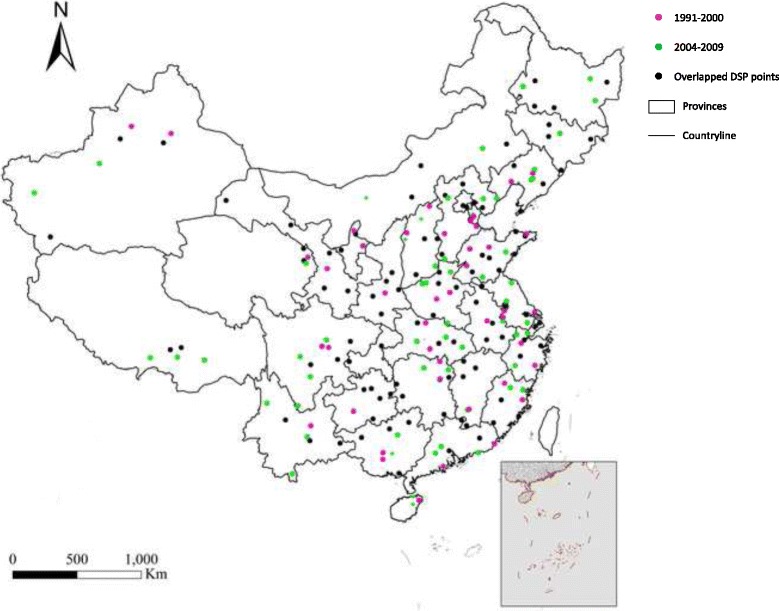
The Map of DSP in different years

The system came almost to a standstill from 2001 to 2003 because of DSP adjustments. So our research analyzed 102 continuous DSP from 1991 to 2000 provided by Institute of Basic Medical Sciences at Chinese Academy of Medical Sciences and from 2004 to 2009 (one point was deleted because of missing data for 5 continuous years) provided by CCDC, covering 7.3 million people in 1991–2000 and expanded to a population of 52 million in the same areas for 2004–2009. Before 2000, 1.3 million subjects were from urban areas and 6 million lived in rural areas. After 2004, due to the strong trend in urbanization, the sample population in the 102 continuous DSP points included 17 million people in urban areas and 35 million in rural areas. When considering the data according to the seven regions, which were created based on the population and area; East areas included the most points, with 26 points that expanded from covering a surveillance population of 2.2 million in the original set to a 13.4 million surveillance population. The Northeast has the fewest points (11) with the lowest population (growing from 0.6 million to 6 million). More details on the characteristics of the 102 DSP points can be found in Additional file 1 and the map of DSP in Fig. 1.

### Data collection and data quality

At each DSP site, there was at least one township hospital, and the ‘Disease Prevention Unit’ in these hospitals was responsible for vital registration. In urban areas, almost half of all deaths occurred in health facilities, and there were standard protocols for death registration that were closely adhered to. The DSP staff collected the death certificate from the hospital, completed by the physician who attended the death. For deaths occurring at home, a staff member from the Unit visited the household, and completed a death certificate based on a description of symptoms from family members (which followed the standardized verbal autopsy procedure), and on available documents from recent contact with health services. The proportion of causes of death by the verbal autopsy (VA) approach was about 7% [13]. Validity of the VA procedure for adult deaths in China has been assessed in another paper [14]. The detailed working procedure for mortality registration is also described in other works [12, 15, 16]. During 1991–1995, about 42% of CVD death cases were diagnosed at hospitals classified as county-level or above [17], and the proportion kept increasing, reaching 56% in 2000 [18]. After 2000, more than 75% of deaths were diagnosed from county-level hospitals or above [19]. The proportion of deaths from cardiovascular diseases diagnosed by clinical and physicochemical means (including blood biochemical examination, electrocardiogram, chest X-ray, even coronary artery Computed Tomography and brain angiography, etc.) was about 60% [19]. In general, the quality of data collection in DSP is high, and this has been validated in other previous studies [20–22].

### Data analysis

#### Redistributing garbage codes

In 1996, the GBD study introduced the term “garbage coding” for the practice of assigning deaths to causes that are not useful for public health analysis of cause-of-death data as part of the assessment of the GBD [23]. This study has used the term garbage code (GC) to refer to all deaths assigned to codes that should be redistributed to enhance the validity of public health analysis. Therefore, certain codes were not regarded as the underlying cause of death, including senility (ICD9–797, ICD10-R54, 1.35%); atherosclerosis (ICD9–440, ICD10-I70, 0.50%); cerebral atherosclerosis (ICD9–437.0, ICD10-I67.2, 0.57%); and chronic pulmonary heart disease (CPHD, ICD9–416, ICD10–127.9, 6.43%). These were labeled garbage. Since the proportion of most garbage codes was small and stable, we did not redistribute them into other categories. The only exception was deaths caused by CPHD, which were classified as having an underlying cause of COPD, since in China 96% of death cases of CPHD developed from COPD [24]. We did not redistribute any other diseases to IHD.

#### Code mapping

The codes for cause of death reported from 1991 to 2000 for DSP data with ICD-9 [25] were shifted to ICD-10 [26] in order to keep them identical and comparable with the causes as classified after 2004. The ICD codes for IHD are 410–414 in ICD-9 and I20-I25 in ICD-10.

### Mortality rate adjustment and analysis

Every three years from 1991 to 2000 and 2006 to 2008, an independent survey based on “capture-mark-recapture” methods was conducted to estimate under-reporting, and mortality estimates were adjusted accordingly [12, 17, 27]. The average under-reporting rates for 1991 to 2000 and 2006 to 2008 were 13% [28] and 17% [27], respectively. Data from DSP in 2004–05 were verified by the national death survey in 2006 [6]. So we used the average rate from the 2006 survey as the rate for 2005. In addition, based on the underreported adjusted rates from 1991 to 2000 and 2006–2009, the envelope of mortality rates for each year were estimated by using the regression model log r_t._ = a + bt, where r_t._ denotes the mortality rate at time point t. Trend b was estimated from the logarithm (log r_t_) of the annual event rates using Poisson Regression [29, 30] In order to verify the increasing and decreasing trends of these diseases, ASDRs for each year were computed based on China’s 2010 census population structure [31].

Trend in IHD was calculated from ASDRs by the joinpoint regression model (desktop version: Version 4.3.1.0 [32]) for the total surveillance population, the male/female categories, the urban/rural categories, and the seven regions. Default maximum number of joinpoints was 2. Monte Carlo simulation, with the number of permutations set to 4499, is used to calculate the permutation *p* value for each hypothesis set. This analysis compared models by starting with no joinpoints and subsequently testing whether 1 or more joinpoints needed to be entered into the model to best fit the data. The most parsimonious models were selected to report the estimated annual percent change (APC) for each time segment detected and the average annual percent change (AAPC) for only 0 joinpoint or for the full study period, along with the estimated models. The terms increasing or decreasing were used to describe the trend when the APC or AAPC was statistically significantly different from 0; otherwise, the term stable was used. All significance tests were 2-sided. Statistical significance was defined as *P* < 0.05.

Most IHD deaths took place after age 40, therefore we restricted the death proportion to ages 40–49, 50–59, 60–69, 70–79 and 80 and over, separately, by sex. Since the surveillance population in each point was not large, the average 5-year (for 1991–1995, 1996–2000), 2-year (for the 2004–2005 survey) or 4-year (for 2006–2009) deaths were used for observation of each DSP point, which helped make numbers more stable.

## Results

**Fig. 2:**
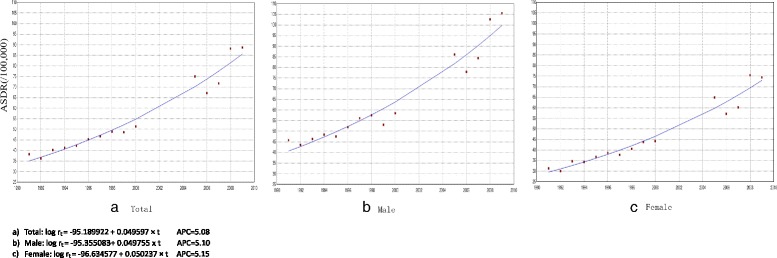
Trend of ASDR of IHD from 1991 to 2009 by sex (/100,000)

The estimation equation on ASDR for IHD by year was log r_t._ = −95.189922 + 0.049597× t, and AAPC was 5.08% with statistical significance. Both male and female mortality rates increased by a similar speed (AAPC = 5%) (Fig. 2). The number of men dying from IHD was consistently higher than the number of women during this time period, which was about 33.3% more male than female deaths annually.

**Fig. 3:**
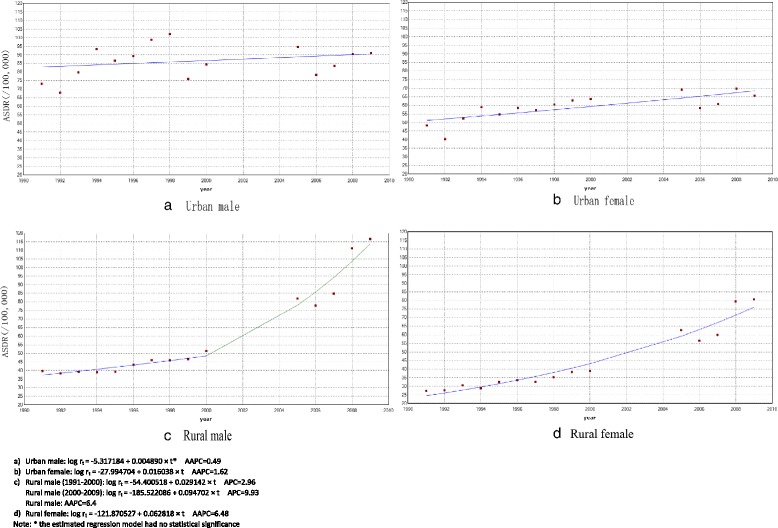
Trend of ASDR for IHD from 1991 to 2009 by sex and urban/rural areas (/100,000)

The increasing trends in urban and rural areas were also statistically significant. However, the increasing trend in urban areas (Urban: log r_t._ = −16.531373 + 0.010404 × t) was not faster than rural areas, with AAPC 1.05% and 6.2%, respectively. In urban areas, the rate for males fluctuated a lot (the estimated regression model had no statistical significance), so the rate for females was responsible for the increase. While in rural areas, the year 2000 was a cut-off point for mortality rate with APC increasing from 3.52% (log r_t._ = −65.372493 + 0.034575 × t) in 1991–2000 to 9.02% (log r_t._ = −168.972238 + 0.086375 × t) in 2000–2009, as well as for male mortality rate (Fig. 3).

**Fig. 4:**
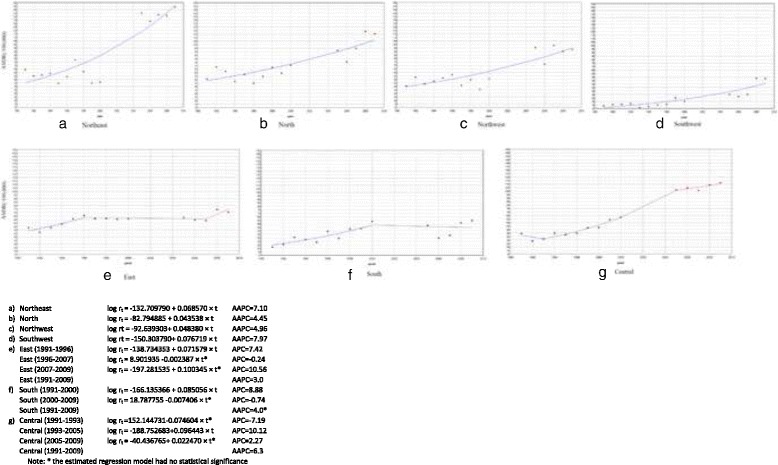
Trend of ASDR for IHD from 1991 to 2009 by 7 regions (/100,000)

In terms of the seven regions, the mortality rates were low in the south areas and high in the north areas. From 1991 to 2009, the mortality rates increased in the Northeast, North, Northwest and Southwest for the whole period, especially in the Southwest (AAPC = 7.97%) and Northeast (AAPC = 7.10%). For the rest of the areas, the mortality rates increased in the East during 1991–1996, in the South during 1991–2000 and in Central areas during 1991–2005 with statistical significance, and showed a stable or decreasing trend for other time periods without statistical significance (Fig. 4).

**Fig. 5:**
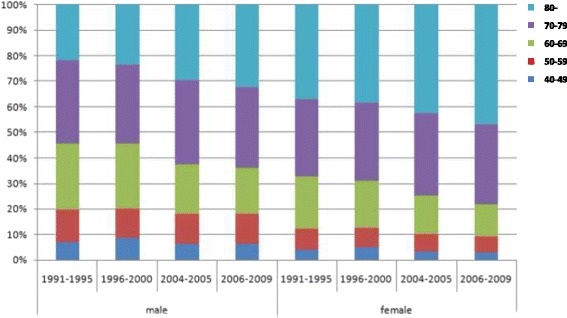
The proportion of deaths above 40 years old for some combined years by sex

For the proportion of deaths after age 40, at ages 40–59 and 70–79, the death proportion remained fairly stable, but the proportion for ages 60–69 reduced with that of ages above 80 years old increasing. Compared to female deaths, more male deaths took place before age 60 (male: about 20%, female: about 10%). For ages 60–69, the male proportion reduced from 25.9% to 17.8% and female reduced from 20.2% to 12.8% from 1991 to 2009 (Fig. 5).

## Discussion

In analyzing areas in China from 1991 to 2009, the ASDR trend for IHD increased substantially for men and women, urban and rural, and in most regions. The speed of increase of ASDR varied greatly between regions of the country and between urban and rural areas. The greater increase in the Northeast and Southwest widened the gap between different regions. Of course, we couldn’t know the situation in other areas in China, if there were an extreme scenario where IHD mortality rate decreased in all other areas in the past 20 years, the trend of IHD mortality rate across the whole country could decrease. But from these 102 continuous DSP, the ASDR trend for IHD should increase.

Actually, the increasing mortality trend for IHD in China matched the related changing levels of prevalence of the various risk factors. In Western countries, it has been shown that IHD is related to high serum total cholesterol (TC) levels [33–35]. With growing urbanization and industrialization in Asia, the same relationship has been shown between TC and IHD [36]. An earlier study focused on Shanghai found a strongly positive, and apparently independent, relation between serum cholesterol concentration and death from IHD [37]. Also a study in Beijing found age-standard IHD mortality increased from 1984 to 1999, which can be explained by rises in TC, reflecting an increasingly “Western” diet [38].

In China, over the past 20 years, cereals decreased as a proportion of the diet, whereas fats increased fast. From 1982 to 2002, fat intake increased from 68.3 g to 85.5 g per day per person in urban areas and increased from 39.6 g to 72.7 g per day per person in rural areas. Although there was an even higher fat intake in urban areas, it increased faster in rural areas [39]. Other studies in Beijing confirmed the trend that the total fat intake increased from 334.5 g/d to 488.4 g/d in urban areas and from 124.8 g/d to 350.7 g/d in rural areas from 1983 to 2002, where it increased faster in rural areas [40, 41]. These diet risk factors give rise to an increased proportion of the population that is overweight and obese. In 2010, the national overweight and obese prevalence for adults was 30.6% and 12.0%, respectively [42], which also caused increasing levels of TC [43]. Thus the TC increase changes the pattern of IHD mortality and morbidity. That’s why the mortality rate in rural areas increased faster.

In addition, in our study, the number of men dying from IHD was higher than the number of women, and also the mortality rate increased faster in Northeast China. This pattern could also be seen in the MONICA study [44] and GBD 2013 [10], which was also associated with risk factors. Hypertension, diabetes, hypercholesterolemia and smoking are major risk factors for IHD [45]. From the 1980s to mid 1990s, blood pressure and overweight values increased significantly, in the north higher than in the south, in males higher than females [46]. And the explanation for more male deaths than female ones could be due to the high levels of tobacco consumption in men (52.1%) compared with women (2.7%) in China [47]. Higher smoking rates were also seen in the northeast and northern parts of China [48].

Jiang, et al. (2011) showed that a large number of IHD patients took place at ages above 60, but the incidence of young people increased [49]. In addition, male IHD incidence rate increased 30.3% among the 35–44 age group and increased 21% among the 45–55 age group within 3 years [50]. From our study, the proportion of deaths at ages 40–59 remained stable with that of ages above 80 years old increasing, which has not shown more and more IHD deaths in young adults as yet, maybe because of medical treatment improvement and life of expectancy of Chinese people increasing. Therefore, not that many young adults died, but the deaths after age 80 could increase.

In this study, since the actual rates fluctuated in some years, the predicted mortality rate for each year calculated by the Poisson regression models was used, instead of the actual rate. This idea comes from GBD research. Before analysis, GBD research usually used models to estimate the envelope to further adjust the mortality rates [51]. Although the predicted mortality for each year would be affected by the mortality or deaths in other years, we think the predicted values are good for trend testing. The data from 102 continuous DSP covered only one street or one block before 2000, and then were expanded to cover a whole district or county, which may have caused some bias. Fortunately, the data collected included a large number of people (7.3 million before the year 2000 and 52 million for 2004–2009) and almost covered 31 out of 33 provinces/municipalities. Furthermore, before year 2000 it was randomly selected from each area and the demographic character (e.g. age structure, lifestyle behavior, etc.) of each point was similar to those after 2000, which means the samples could represent the whole and are relevant for comparison. So we can assume these samples, coming from the relative fixed population or the same primary sampling units (PSUs), should have the lowest variance for estimated difference for a variable of interest from repeated surveys from a statistical point of view.

In another paper we already found the reason that caused the trend decrease in GBD 2013 - chronic pulmonary heart disease as GC redistribution [52]. Before 2000, in the mortality database of DSP, there was a corresponding proportion of chronic PHD. In GBD 2010, the death cases of chronic PHD were totally redistributed into COPD, which was the same as the results from the original methods in the DSP system, as well as this study. But the cases were redistributed into COPD, IHD and hypertension heart disease by different fractions based on the module in GBD 2013. COPD is the major cause of chronic PHD, and probably accounts for 80–90% of the causes from western literature [53]. Studies in China testified that the proportion was even higher for Chinese. Fu et al. (1988) investigation confirmed that 96.6% of Chronic PHD was from COPD in Beijing [24]. Owing to changing the redistribution fraction of chronic PHD, more IHD cases were adjusted in the early 1990s. So that’s why GBD 2010 and our study’s results showed that the mortality trend increased, while GBD 2013 results showed the trend remained stable. Therefore, in GBD 2015, using the same GC redistribution strategy of GBD 2010, the trend increased in China. So in a nutshell, the increased mortality trend for IHD could describe the real world for Chinese people. From this paper, it is indicated that in the past two decades the ASDR trend for IHD has increased by an estimated annual 1.2 million deaths, which has caused a challengeable burden of diseases. Therefore, control of risk factors in China is an urgent task, including controlling tobacco use and promoting healthy diets, which could lower death rates and avoid increased medical expense.

### Limitations

While this paper utilizes extensive and wide-ranging data, this data does have some limitations. The data used in this paper only covers 1991 to 2009 and thus does not reveal more recent trends or patterns. However, the time period studied captures the critical period of transition in Chinese society and the economy. The data from continuous DSPs covered only one street or one block before 2000, and then were expanded to cover a whole district or county; hence a sensitivity analysis should be done. As a result, most of the cases of death’s home address was not described in detail, so it is hard for us to identify those cases of deaths whose home address was in the streets or blocks which were both active in both periods. That’s one of the limitations in this study. Another limitation in this study is that we did not take the sampling weights and strata into account.

## Conclusions

By observing a wide range of areas across China from 1991 to 2009, this paper concludes that the ASDR trend for IHD has increased. These trends reflect changes in the Chinese standard of living and lifestyle with diets higher in fat, higher blood lipids and increased body weights. Therefore, control of risk factors in China is an urgent task to lower IHD death rates and avoid increased medical expense.

## Additional file

Additional file 1: The Characteristics of 102 DSP Points. There were four variables in this table, including the DSPs name, regions where each DSP belongs to, population for each DSP in 1991 and 2009 respectively. (DOCX 49 kb)

## Abbreviations

AAPC: Average Annual Percentage Change
APC: Annual Percent Change
ASDRs: Age-standard Death Rates
CCDC: Chinese Center for Disease Control & Prevention
COPD: Chronic Obstructive Pulmonary Diseases
CPHD: Chronic Pulmonary Heart Disease
CVD: Cardiovascular Disease
DSP: Disease Surveillance Points
GBD: Global Burden of Disease
GC: Garbage Code
IHD: Ischemic Heart Disease
NHFPC: National Health and Family Planning Commission
PSUs: Primary Sampling Units
TC: Total Cholesterol
VA: Verbal Autopsy
VR: Vital Registration
