# Supplementary material for: Mortality trends for ischemic heart disease in China: an analysis of 102 continuous disease surveillance points from 1991 to 2009
Source: BMC Public Health. 2017 Jul 25;18:52. doi: 10.1186/s12889-017-4558-3 (PMC5526279; doi:10.1186/s12889-017-4558-3)
Supplement: Additional file 1: — The Characteristics of 102 DSP Points. There were four variables in this table, including the DSPs name, regions where each DSP belongs to, population for each DSP in 1991 and 2009 respectively. (DOCX 49 kb) [file 12889_2017_4558_MOESM1_ESM.docx]

**Appendix 1: The Characteristics of 102 DSP Points**

Appendix1: The Characteristics of 102 DSP Points

| **DSPs** | **Region** | **Urban/Rural** | **Population**  **in 1991** | **Population**  **in 2009** |
| --- | --- | --- | --- | --- |
| Dongcheng District, Beijing | North | urban | 61,212 | 689,006 |
| Tongzhou District, Beijing | North | rural(urban)* | 36,456 | 649,234 |
| Ji County, Tianjin | North | rural | 13,957 | 965,109 |
| Kaiping District, Tangshan City, Hebei Province | North | urban | 39,882 | 272,989 |
| Wu'an City, Hebei Province | North | rural | 103,160 | 754,904 |
| Qiaodong District, Zhangjiakou City, Hebei Province | North | urban | 34,785 | 213,495 |
| Fengning County, Hebei Province | North | rural | 105,560 | 350,036 |
| Xinghualing District, Taiyuan City, Shanxi Province | North | urban | 22,892 | 556,731 |
| Pingding County, Shanxi Province | North | rural | 50,452 | 338,511 |
| Jiang County, Shanxi Province | North | rural | 112,847 | 280,031 |
| Humin District, Huhehaote City, Inner Mongolia | North | urban | 49,792 | 220,952 |
| Kailu County, Inner Mongolia | North | rural | 15,800 | 405,414 |
| Suniteyou County, Inner Mongolia | North | rural | 8,044 | 58,326 |
| Shahekou District, Dalian City, Liaoning Province | Northeast | urban | 47,504 | 656,640 |
| Fengcheng County, Liaoning Province | Northeast | rural | 54,557 | 579,506 |
| Fuxin County, Liaoning Province | Northeast | rural | 69,141 | 751,386 |
| Nanguan District, Changchun City, Jilin Province | Northeast | urban | 48,806 | 357,842 |
| Dehui County, Jilin Province | Northeast | rural | 49,960 | 907,087 |
| Ji'an District, Jilin Province | Northeast | rural | 111,927 | 238,132 |
| Longjin District, Jilin Province | Northeast | urban (rural)* | 19,744 | 204,084 |
| Nangang District, Ha'erbin City, Heilongjiang Province | Northeast | urban | 51,056 | 988,862 |
| Yi'an County, Heilongjiang Province | Northeast | rural | 154,213 | 480,508 |
| Baoqing County, Heilongjiang Province | Northeast | rural | 80,957 | 412,456 |
| Datong District, Daqing City, Heilongjiang Province | Northeast | urban | 23,448 | 245,780 |
| Luwan District, Shanghai | East | urban | 82,544 | 308,742 |
| Songjiang County, Shanghai | East | rural(urban)* | 60,678 | 741,878 |
| Yunlong District, Xuzhou City, Jiangsu Province | East | urban | 47,575 | 238,561 |
| Zhangjiagang City, Jiangsu Province | East | rural | 146,661 | 1,029,030 |
| Xiacheng District, Hangzhou City, Zhejiang Province | East | urban | 54,704 | 359,795 |
| Fenghua County, Zhejiang Province | East | rural | 103,565 | 524,496 |
| Jinhua City, Zhejiang Province | East | urban | 59,849 | 468,415 |
| Anqing City, Anhui Province | East | urban | 62,938 | 176,444 |
| Tianchang County, Anhui Province | East | rural | 114,317 | 604,931 |
| Caohu City, Anhui Province | East | rural(urban)* | 154,718 | 801,724 |
| Mengcheng City, Anhui Province | East | rural | 173,297 | 1,173,376 |
| Jing County, Anhui Province | East | rural | 54,633 | 338,658 |
| Meilie District, Sanming City, Fujian Province | East | urban | 51,802 | 159,676 |
| Hui'an County, Fujian Province | East | rural | 106,288 | 976,938 |
| Yongding County, Fujian Province | East | rural | 118,167 | 430,582 |
| Donghu District, Nanchang City, Jiangxi Province | East | urban | 18,912 | 403,738 |
| Wuning County, Jiangxi Province | East | rural | 115,925 | 375,781 |
| Longlan County, Jiangxi Province | East | rural | 92,614 | 308,035 |
| Shanggao County, Jiangxi Province | East | rural | 38,732 | 358,986 |
| Shibei District, Qingdao City, Shandong Province | East | urban | 35,959 | 537,819 |
| Linyuan County, Shandong Province | East | rural | 98,997 | 560,479 |
| Yantai City, Shandong Province | East | urban | 28,737 | 698,968 |
| Penglai County, Shandong Province | East | rural | 67,278 | 411,744 |
| Jining City, Shandong Province | East | rural(urban)* | 110,252 | 212,431 |
| Laiwu City, Shandong Province | East | rural(urban)* | 114,687 | 1,037,132 |
| Lunan County, Shandong Province | East | rural | 95,810 | 1,001,486 |
| Zhongyuan District, Zhengzhou City, Henan Province | Central | urban | 30,594 | 489,458 |
| Huxian City, Henan Province | Central | rural | 96,301 | 803,106 |
| Tanghe County, Henan Province | Central | rural | 127,596 | 1,193,603 |
| Zhui County, Henan Province | Central | rural | 120,377 | 781,843 |
| Jiang'an District, Wuhan City, Hubei Province | Central | urban | 62,933 | 710,219 |
| Gucheng County, Hubei Province | Central | rural | 78,151 | 526,363 |
| Tianmen City, Hubei Province | Central | rural | 126,464 | 1,552,561 |
| Liuyang City, Hunan Province | Central | rural | 161,326 | 1,322,418 |
| Chenzhou City, Hunan Province | Central | rural(urban)* | 96,730 | 343,578 |
| Hongjiang City, Hunan Province | Central | rural | 116,748 | 456,244 |
| Fenghuang County, Hunan Province | Central | rural | 125,588 | 347,712 |
| Yuexiu District, Guangzhou City, Guangdong Province | South | urban | 43,730 | 1,163,751 |
| Nanxiong County, Guangdong Province | South | rural | 144,017 | 411,378 |
| Wuhua County, Guangdong Province | South | rural | 94,470 | 991,914 |
| Bingyang County, Guangxi Province | South | rural | 187,639 | 936,500 |
| Guilin City, Guangxi Province | South | urban | 14,467 | 109,795 |
| Hepu County, Guangxi Province | South | rural | 35,741 | 944,218 |
| Lingyun County, Guangxi Province | South | rural | 21,494 | 195,064 |
| LuochengMulao Race District, Guangxi Province | South | rural | 131,084 | 354,584 |
| Zhengdong District, Haikou City, Hainan Province | South | urban（rural）* | 80,360 | 393,580 |
| Wanzhou City, Chongqing | Southwest | urban | 36,658 | 1,517,151 |
| Dazu County, Chongqing | Southwest | rural | 112,144 | 850,104 |
| Zizhong County, Sichuan Province | Southwest | rural | 200,702 | 1,273,725 |
| Xichong County, Sichuan Province | Southwest | rural | 90,719 | 562,141 |
| Hanyuan County, Sichuan Province | Southwest | rural | 106,252 | 344,603 |
| Honghuagang District, Zunyi City, Guizhou Province | Southwest | urban | 13,920 | 496,594 |
| Meitan County, Guizhou Province | Southwest | rural | 63,253 | 438,202 |
| Yuping County, Guizhou Province | Southwest | rural | 120,543 | 131,971 |
| Shibing County, Guizhou Province | Southwest | rural | 68,344 | 146,143 |
| Dushan County, Guizhou Province | Southwest | rural | 32,151 | 338,698 |
| Tonghai County, Yunnan Province | Southwest | rural | 70,185 | 288,960 |
| Guangnan County, Yunnan Province | Southwest | rural | 115,057 | 796,273 |
| Xiangyun County, Yunnan Province | Southwest | rural | 190,017 | 465,321 |
| Lhasa City, Tibet | Southwest | urban | 13,663 | 239,072 |
| [Maizhokunggar County, Tibet](http://www.baidu.com/link?url=vXsEK4jKQL8wjAl8Soj9xA0au7QpZ4C3GmKpzIrJYzng9ERqJp_37UEzdO5vU7aWAfuxFQr_cuHtARzcaBiC-MsHXtM5WbzMPohRjo2AL6W) | Southwest | rural | 35,436 | 42,632 |
| Tongzhou City, Shaanxi Province | Northwest | urban | 11,758 | 199,649 |
| Mei County, Shaanxi Province | Northwest | rural | 89,830 | 308,479 |
| Huayin County, Shaanxi Province | Northwest | rural | 59,397 | 259,285 |
| Luochuan County, Shaanxi Province | Northwest | rural | 110,213 | 217,931 |
| Hanyin County, Shaanxi Province | Northwest | rural | 23,942 | 248,208 |
| Jintai County, Gansu Province | Northwest | rural | 67,128 | 238,716 |
| Beidao District, Tianshui City, Gansu Province | Northwest | rural(urban)* | 40,901 | 573,931 |
| Zhangye City, Gansu Province | Northwest | rural | 27,401 | 516,262 |
| Dunhuang City, Gansu Province | Northwest | rural | 24,004 | 136,460 |
| Lintan County, Gansu Province | Northwest | rural | 24,484 | 147,416 |
| Chengzhong District, Xining City, Qinghai Province | Northwest | urban | 26,825 | 170,261 |
| Menyuan County, Qinghai Province | Northwest | rural | 15,999 | 164,838 |
| Yinchuan City, Ningxia Province | Northwest | urban | 24,151 | 421,305 |
| Zhongwei County, Ningxia Province | Northwest | rural | 5,347 | 362,070 |
| Tianshan District, Wurumuqi City, Xinjiang Province | Northwest | urban | 24,565 | 534,935 |
| Hetian County, Xinjiang Province | Northwest | rural | 39,230 | 307,714 |
| Xinyuan County, Xinjiang Province | Northwest | rural | 19,523 | 321,280 |

Note: *After 2005, the classification of these districts was changed from urban to rural, or from rural to urban.
